# A Direct n^+^-Formation Process by Magnetron Sputtering an Inter-Layer Dielectric for Self-Aligned Coplanar Indium Gallium Zinc Oxide Thin-Film Transistors

**DOI:** 10.3390/mi13050652

**Published:** 2022-04-19

**Authors:** Xinlv Duan, Congyan Lu, Xichen Chuai, Qian Chen, Guanhua Yang, Di Geng

**Affiliations:** 1 Key Laboratory of Microelectronics Device and Integrated Technology, Institute of Microelectronics of Chinese Academy of Sciences, Beijing 100029, China; duanxinlv@ime.ac.cn (X.D.); lucongyan@ime.ac.cn (C.L.); chuaixichen@ime.ac.cn (X.C.); chenqian@ime.ac.cn (Q.C.); yangguanhua@ime.ac.cn (G.Y.); 2 University of Chinese Academy of Sciences, Beijing 100049, China

**Keywords:** self-aligned coplanar, IGZO TFT, S/D region, n^+^-formation, magnetron sputtering inter-layer dielectric

## Abstract

An inter-layer dielectric (ILD) deposition process to simultaneously form the conductive regions of self-aligned (SA) coplanar In-Ga-Zn-O (IGZO) thin-film transistors (TFTs) is demonstrated. N^+^-IGZO regions and excellent ohmic contact can be obtained without additional steps by using a magnetron sputtering process to deposit a SiO_x_ ILD. The fabricated IGZO TFTs show a subthreshold swing (SS) of 94.16 mV/decade and a linear-region field-effect mobility (*μ_FE_*) of 23.06 cm^2^/Vs. The channel-width-normalized source/drain series resistance (*R_SD_W*) extracted using the transmission line method (TLM) is approximately as low as 9.4 Ω·cm. The fabricated ring oscillator (RO) with a maximum oscillation frequency of 1.75 MHz also verifies the applicability of the TFTs.

## 1. Introduction

**Figure 1:**
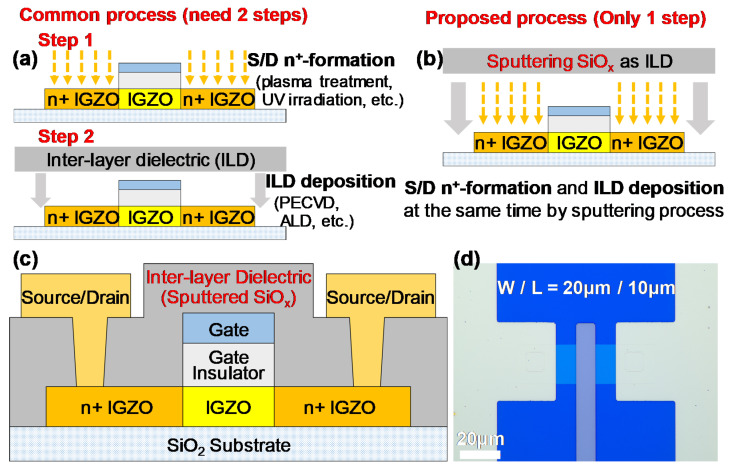
(**a**) Common two-step process and (**b**) proposed one-step process for IGZO n^+^-region formation and ILD deposition. (**c**) Cross-sectional diagram and (**d**) optical microscope image of the fabricated SA coplanar IGZO TFT.

Indium-Gallium-Zinc-Oxide (IGZO) thin-film transistors (TFTs) with staggered structures, such as etch-stopper (ES) and back-channel-etched (BCE) structures, have been proven to be useable as circuit devices in flat-panel displays [1,2]. However, due to the overlap between gate and source/drain (S/D) electrodes, these staggered-structure devices inevitably have large parasitic capacitances, which result in a low operating speed of TFT devices. A self-aligned (SA) coplanar structure is a promising solution to overcome this parasitic capacitance problem [3]. Forming conductive n^+^-IGZO to obtain ohmic contact between active S/D regions and S/D electrodes is an important process for SA coplanar devices. Many methods for this process have been proposed, and the fabricated IGZO devices have good performance. Plasma treatment (Ar, H_2_, etc.) [4,5] and deep-ultraviolet (DUV) irradiation [6] are commonly used. However, these solutions require an extra step, as shown in Figure 1a, which leads to additional process costs. Forming n^+^-IGZO during the overetching of a SiO_2_ gate insulator (GI) is a simple method [7,8]. However, this method is not applicable when the GI-etching plasma can etch IGZO films. Recently, the formation of n^+^-IGZO regions by simply coating an organic inter-layer dielectric (ILD) has been demonstrated, and a channel-width-normalized S/D series resistance (*R_SD_W*) of 24 Ω·cm was obtained [9]. This report shows the possibility of forming n^+^-IGZO regions during the ILD deposition process. Based on this idea, other novel methods to fabricate low-*R_SD_W* SA coplanar IGZO TFTs are worth investigating.

In this work, we use a magnetron sputtering process to deposit a SiO_x_ ILD and simultaneously form n^+^-IGZO regions for SA coplanar IGZO TFTs. In this way, ILD deposition and n^+^-formation can be combined into one step, as shown in Figure 1b. The fabricated devices have quite low *R_SD_W*. The mechanism of reducing the IGZO film resistivity by the sputtering process is investigated by X-ray photoelectron spectroscopy (XPS) analysis. Ring oscillators (ROs) composed of the fabricated SA coplanar TFTs show good frequency characteristics, which indicates that these TFTs have the potential to be used in high-speed circuits. SiO_x_ is a commonly used insulating layer [10]; moreover, sputtering SiO_x_ and sputtering IGZO can share one piece of sputtering equipment. Considering the material compatibility and equipment compatibility of sputtered SiO_x_, as well as the reduction in process steps, this sputtering treatment method is expected to become an industrial production technology for SA coplanar IGZO TFTs.

## 2. Experiment

SA coplanar IGZO TFTs are fabricated on a 300-nm-thick thermal silicon dioxide substrate. First, an IGZO film with a thickness of 20 nm is deposited as an active layer by a magnetron sputtering process using an IGZO target with an atomic ratio of In_2_O_3_:Ga_2_O_3_:ZnO = 1:1:2 mol.%. After depositing 30-nm-thick Al_2_O_3_ as a GI by atomic layer deposition (ALD), a 50/50-nm-thick Ti/Cr bilayer is formed as a gate electrode by a lift-off process. By using the gate electrode as a shield layer, the GI can be SA-etched by Ar/BCl_3_ (10 sccm/40 sccm) plasma etching [11] without an additional photolithography step. This plasma etching must be precisely controlled to avoid etching the IGZO layer since Ar/BCl_3_ plasma can also etch IGZO films [12]. Diluted acid is used as a wet etchant for patterning the IGZO layer [13]. Then, a SiO_x_ ILD layer with a thickness of 50 nm is deposited by a magnetron sputtering process under an Ar:O_2_ gas ratio of 20:1 sccm, and, simultaneously, the n^+^-formation process of the S/D region in the IGZO layer is completed. The SiO_x_ layer is etched by a dry etching process to form contact holes. A 20/80-nm Ti/Au bilayer is deposited as S/D electrodes and patterned by a lift-off process. Finally, a postannealing process is performed at a temperature of 180 °C for 2 h.

Figure 1c shows a cross-sectional diagram of the fabricated devices. Figure 1d shows an optical microscope image of a fabricated device with a channel width (*W*) of 20 μm and a channel length (*L*) of 10 μm.

## 3. Results and Discussion

**Figure 2:**
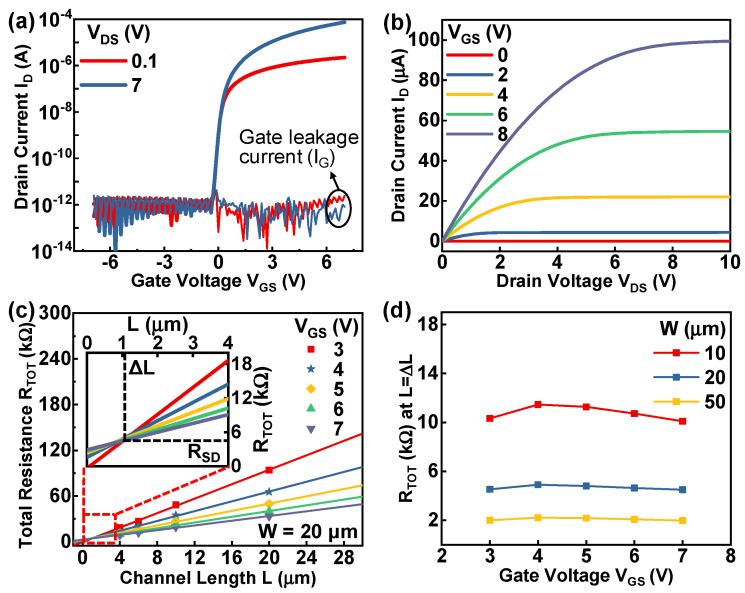
(**a**) Transfer characteristics and (**b**) output characteristics of a fabricated device. (**c**) Variation in the total resistance (*R_TOT_*) as a function of channel length (channel width is fixed at 20 μm) at various gate voltages. (**d**) *R_TOT_* values at *L* = Δ*L* of the TFTs with different channel widths extracted using the transmission line method.

The fabricated SA coplanar IGZO TFTs are measured using an Agilent B1500A semiconductor parameter analyzer in a dark box. Figure 2a shows the transfer characteristics and gate leakage characteristics of the TFT with *W* = 10 μm and *L* = 15 μm at drain voltages (*V_DS_*) of 0.1 and 7 V. The transfer curves exhibit excellent device performance with a low gate leakage current characteristic. The transistor parameters of the device presented in Figure 2a can be estimated from the linear-region transfer curve (*V_DS_* = 0.1 V). The turn-on voltage (V_on_) and subthreshold swing (SS) are estimated to be −0.3 V and 94.16 mV/decade, respectively. The field-effect mobility (*μ_FE_*) can be calculated from the transfer curve in the linear region (*V_DS_* = 0.1 V) using the following equation [6]:

(1) $$\mu_{FE}=\frac{{Lg}_{m}}{{WC}_{i}V_{DS}}=\frac{{L(dI}_{D}{/dV}_{GS})}{{WC}_{i}V_{DS}}$$

where *L* is the channel length, *g_m_* is the transconductance, *I_D_* is the drain current, *V_GS_* is the gate voltage, *W* is the channel width, *C_i_* is the capacitance per unit area of gate oxide, and *V_DS_* is the drain bias (0.1 V). According to this equation, the calculated *μ_FE_* of the presented device is approximately 23.06 cm^2^/Vs. In addition, the average *μ_FE_* and SS of the 37 devices under test (DUTs) are approximately 21.37 cm^2^/Vs and 100.35 mV/decade, respectively. Figure 2b shows the output characteristics of the same TFT under varying gate voltage (*V_GS_*) from 0 V to 8 V. The output curves exhibit a good saturation characteristic at large *V_DS_* and good ohmic behavior at low *V_DS_*, which means that the IGZO film of the fabricated device has good ohmic contact with the S/D electrodes.

The S/D series resistance (*R_SD_*) is extracted by using the transmission line method (TLM) [6,14]. In this method, the total resistance (*R_TOT_*) of a fabricated device is given by *V_DS_*/*I_DS_* from the transfer curve in the linear region. *R_TOT_* can be expressed by the following equation [6]:

(2) $$R_{TOT}=\frac{V_{DS}}{I_{DS}}{=R}_{ch}{+R}_{SD}=\frac{L-\Delta L}{\mu_{FE}C_{i}W\left(V_{GS}\;{-V}_{th}\right)}{+R}_{SD}$$

where *R_ch_* is the channel resistance, *L* is the designed channel length, Δ*L* is the difference between the designed channel length and the actual channel length, *C_i_* is the capacitance per unit area of gate oxide, *W* is the channel width, and *V_th_* is the threshold voltage. According to this equation, when *L −* Δ*L* = 0, *R_SD_* equals *R_TOT_*. Therefore, by fitting the *R_TOT_*–*L* relationship of the TFTs with a fixed *W* and varying *V_GS_*, the fitted lines will intersect at the point of *L −* Δ*L* = 0, and the y-axis value of this point will be *R_SD_*. Figure 2c shows the *R_TOT_*–*L* relationship of the fabricated devices with a fixed W of 20 μm at V_DS_ = 0.1 V. The intersection is defined as the point where the *R_TOT_* variance of the fitted lines is minimal, and the mean value of the corresponding *R_TOT_* is estimated as *R_SD_*. Details near the intersection are shown as the inset in Figure 2c. *R_SD_* and Δ*L* are evaluated to be 4.68 kΩ and 1.03 μm, respectively. The *R_TOT_* values under different *V_GS_* at *L* = Δ*L* (Δ*L* = 1.03 μm when *W* = 20 μm) are shown as the blue line in Figure 2d. Since the R_SD_ value is related to W [15], the R_SD_ values of the fabricated devices with *W* of 10 μm and 50 μm are also extracted using the TLM, and the corresponding R_TOT_ at *L* = Δ*L* (Δ*L* = 1.23 μm when *W* = 10 μm and Δ*L* = 0.88 μm when *W* = 50 μm) are shown as the red line and yellow line in Figure 2d, respectively. According to Figure 2d, the *R_SD_* values obtained from the mean value of the corresponding *R_TOT_* are 10.77 kΩ and 2.09 kΩ when *W* = 10 μm and 50 μm, respectively. The channel-width-normalized *R_SD_* (*R_SD_W*) is obtained by the product of *R_SD_* and *W* [15]. Therefore, the *R_SD_W* values of the TFTs with *W* = 10 μm, 20 μm, and 50 μm are approximately 10.8 Ω·cm, 9.4 Ω·cm, and 10.5 Ω·cm, respectively, and the mean value of these three *R_SD_W* values is the average *R_SD_W*, which is 10.2 Ω·cm. At different *W*, the *R_SD_W* values are similar and quite small, which proves that the extracted *R_SD_W* values are convincing, and the fabricated TFTs have good ohmic contacts with good uniformity. Table 1 shows the comparison of the *R_SD_W* extracted from IGZO TFTs with different n^+^-formation processes. The *R_SD_W* value of this work is the smallest among these works, which further proves that the excellent ohmic properties of the fabricated TFTs in this work are competitive in the field of IGZO TFTs.

**Figure 3:**
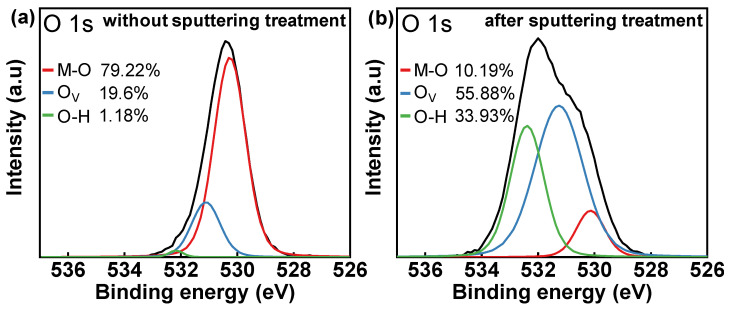
XPS spectra of IGZO films showing O 1s peaks in different states. (**a**) Without sputtering treatment. (**b**) After sputtering treatment.

The mechanism of reducing the IGZO resistivity by sputtering treatment is also investigated using XPS analysis. The O 1s peaks of IGZO films without and after sputtering treatment are shown in Figure 3a,b, respectively. The peak is fitted by three Gaussian distributions, centered at the low binding energy peak (O_L_) of 530.15 eV, the medium binding energy peak (O_M_) of 531.25 eV, and the high binding energy peak (O_H_) of 532.4 eV. O_L_ is related to the oxygen in the metal-oxide bond (M-O), which forms the stable amorphous structure of IGZO films [18]. O_M_ can be assigned to oxygen vacancies (O_V_), which are generally considered to be donor defects [6,19]. O_H_ is commonly attributed to the oxygen in hydroxide (O-H), which is associated with shallow donors [6,18,20]. Compared to the 19.6% concentration of O_V_ in the untreated IGZO film in Figure 3a, the O_V_ concentration of the IGZO film after sputtering treatment significantly increases to 55.88%. The dissociation energies of Si-O, Ga-O, In-O, and Zn-O bonds are 799 kJ/mol, 374 kJ/mol, 346 kJ/mol, and <250 kJ/mol, respectively [21]. During the sputtering deposition of the SiO_x_ ILD, the ion bombardment breaks the M-O bonds in the IGZO film. Since the oxygen content in the sputtering atmosphere is very low (<4 mol%) and the Si-O bond exhibits a larger dissociation energy, Si may take away the oxygen ions in the IGZO film. This process can be represented by the following equation:

SiO_x_ + InGaZnO_4_ → SiO_x+n_ + InGaZnO_4-n_ + n × O_V_ (3)

This reaction could be the mechanism for the increase in the O_V_ concentration after sputtering deposition. Generally, O_V_ are considered to act as donor defects in IGZO films that supply electron carriers for conduction [19,22], and an increase in the O_V_ concentration in IGZO films usually means high conductivity. Therefore, sputtering SiO_x_ as the ILD of SA coplanar IGZO TFTs can effectively form ohmic contact between the IGZO-S/D region and the S/D electrode.

**Figure 4:**
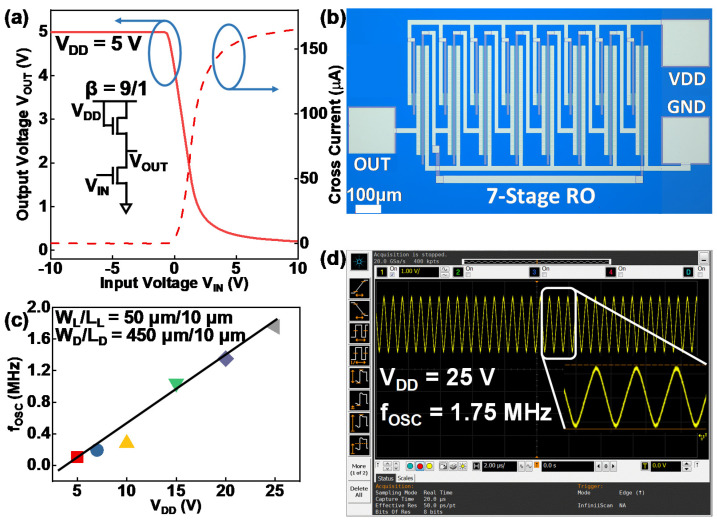
(**a**) VTC, cross current, and schematic of the inverter with a β of 9/1 at V_DD_ = 5 V. (**b**) Optical microscope image of the fabricated 7-stage RO. (**c**) RO frequency under different V_DD_. (**d**) RO output at V_DD_ = 25 V measured by an oscilloscope.

To demonstrate the usability of the proposed SA coplanar IGZO TFTs, inverters and ROs consisting of the fabricated devices are also measured [23,24]. Figure 4a shows the voltage transfer characteristic (VTC) and corresponding cross current of the inverter with a beta ratio (β) of 9/1. Specifically, the W/L ratios of the load TFT (W_L_/L_L_) and driving TFT (W_D_/L_D_) are 50 μm/10 μm and 450 μm/10 μm, respectively. The inset of Figure 4a shows a schematic of the inverter. This inverter shows good level conversion at a supply voltage (V_DD_) of 5 V. Figure 4b shows an optical microscope image of the fabricated seven-stage RO with a buffer inverter. The buffer inverter is connected to the “OUT” pad for measuring the output signal of the RO. During the measurement, a constant voltage signal of different values is applied on the “VDD” pad, and the “GND” pad is connected to a ground signal. An oscilloscope is used to detect the output signal through the “OUT” pad. Figure 4c shows the RO frequency (f_OSC_) measured under different V_DD_ varying from 5 V to 25 V. As V_DD_ increases, f_OSC_ increases accordingly. When V_DD_ = 5 V, the measured f_OSC_ is 105.8 kHz. A maximum f_OSC_ of 1.75 MHz is measured when V_DD_ = 25 V. According to Figure 4c, the frequency of the fabricated RO exhibits a linear correlation with the supply voltage. Figure 4d shows the waveform of the output signal with a maximum frequency of 1.75 MHz detected by the oscilloscope. The inset of Figure 4d shows the waveform after zooming in. The smooth transition between high level and low level observed from the waveform indicates that the measured RO can work stably, which means that the fabricated TFTs are suitable for the application of high-speed IGZO TFT circuits.

## 4. Conclusions

A direct n^+^-region formation process for SA coplanar IGZO TFTs is proposed and studied. By employing magnetron sputtering to both deposit the SiO_x_ ILD layer and reduce the resistivity of the IGZO S/D regions, ohmic contact between the IGZO layer and the S/D metal electrode can be simply obtained without additional steps or equipment. The fabricated TFTs exhibit excellent performance, with V_on_, SS, and linear-region μ_FE_ of −0.3 V, 94.16 mV/decade, and 23.06 cm^2^/Vs, respectively. By using the TLM, the extracted minimum *R_SD_W* is approximately 9.4 Ω·cm. XPS analysis reveals that the improved conductivity of IGZO films can be attributed to the significant increase in O_V_ concentration. The fabricated inverter shows good level conversion. The measured maximum f_OSC_ of the RO output waveform can reach 1.75 MHz with a smooth transition. Because of the process compatibility and excellent device performance, the fabrication technology proposed in this work is expected to be applied in the production of high-speed TFT circuits and flat-panel displays.

## Figures and Tables

**Table 1:** Benchmark of minimum *R_SD_W* for IGZO TFTs with different n^+^-formation processes.

Reference	n^+^-Formation Process	*R_SD_W*_min_ (Ω·cm)
[8]	Overetch SiO_2_ GI	21.8
[9]	Organic ILD	24
[16]	H_2_ plasma treatment	75.5
[5]	Ar plasma treatment	128
[17]	UV irradiation	27
**This work**	**Sputtered ILD**	**9.4**

## Data Availability

The data that support the findings of this study are available from the corresponding author upon request.
